# Differences in Colour Pattern, Behaviour and Gene Expression in the Brain Suggest Divergent Camouflage Strategies in Sympatric Reef Fish Species

**DOI:** 10.1111/mec.17748

**Published:** 2025-04-29

**Authors:** M. J. Heckwolf, J. Gismann, M. González‐Santoro, F. Coulmance, J. Fuß, W. O. McMillan, O. Puebla

**Affiliations:** ^1^ Leibniz Center for Tropical Marine Research (ZMT) Bremen Germany; ^2^ Smithsonian Tropical Research Institute (STRI) Panama Republic of Panama; ^3^ Groningen Institute for Evolutionary Life Sciences (GELIFES) University of Groningen Groningen the Netherlands; ^4^ Department of Biological Sciences University of Pittsburgh Pittsburgh Pennsylvania USA; ^5^ Institute for Chemistry and Biology of the Marine Environment (ICBM), Carl von Ossietzky Universität Oldenburg Oldenburg Germany; ^6^ Institute of Clinical Molecular Biology, Christian Albrechts University of Kiel Kiel Germany

**Keywords:** behaviour/social evolution, molecular evolution, predator prey interactions, speciation, transcriptomics

## Abstract

Camouflage is a critical survival strategy that helps to evade predation and increase hunting success. Background matching and disruptive colouration are different camouflage strategies that are subject to different selective pressures and can drive divergence in their associated traits such as colour pattern and behaviour. This study tested whether two closely related reef fish species (*Hypoplectrus* spp.) with distinct colour patterns exhibit different predator escape responses and differential gene expression in the brain indicative of divergent camouflage strategies. Combining field and laboratory experiments, we show that barred hamlets, characterised by disruptive colouration, are dynamic in their escape responses, while black hamlets, with their darker colouration, had a preference for hiding. The behavioural differences between these species seem to be limited to divergent predator escape responses since other behaviours such as activity or sociability did not differ. Importantly, the observed behavioural differences were accompanied by transcriptomic differences in their brains, particularly in regions associated with the perception of looming threats and less so in the region involved in conditioning. Differential expression in the diencephalon suggests enhanced neuronal plasticity in barred hamlets, which might allow for rapid adjustments in their escape response, while black hamlets exhibited upregulation in genes linked to immune response and oxygen transport in the optic tectum. Overall, our findings suggest that the two species utilise different camouflage strategies, which might contribute to the maintenance of colour pattern differences and thereby influence the speciation and diversification of these closely related sympatric reef fishes.

## 1 Introduction

Colouration and patterning are rapidly evolving traits that serve various functions, including camouflage, mimicry and intra‐ and interspecific communication (McMillan et al. 1999; Phillips et al. 2017; Randall 2005; Tibblin et al. 2020). Inherently, they are shaped by ecological interactions like predation, hunting success, mate choice and competition, which subject them to both natural and sexual selection. One of the most important evolutionary forces driving colouration and patterning appears to be camouflage, a mechanism through which animals blend in with their environment to avoid predation or to increase hunting success (Akkaynak et al. 2017; Caro 2005; Shavit et al. 2023). By avoiding detection, prey can evade predation and save energy that would otherwise be spent on escaping, which gives them a considerable fitness advantage (MøLLER et al. 2019; Stevens and Merilaita 2011; Troscianko et al. 2016). Depending on the species' environment and phenotypic characteristics, camouflage can take several forms, such as background matching and disruptive colouration (Cuthill et al. 2005; Endler 2006; Merilaita and Lind 2005). In background matching, an organism's shape, colour and pattern mimic its surroundings, allowing it to avoid detection by blending seamlessly with its habitat (Merilaita and Stevens 2011). Conversely, disruptive colouration employs contrasting colours and patterns at the body periphery to obscure its shape or outline (Cuthill et al. 2005). These distinct markings can be bars or spots that distort the appearance of the animal when seen from a distance (Stevens and Merilaita 2009).

The effectiveness of camouflage does not only depend on colour pattern but also on accompanying behaviours, such as background choice, body positioning and movement strategy. These behaviours can be triggered through self‐assessment or can be genetically encoded, which allows them to co‐evolve with colour patterns in response to predator–prey interactions (Camacho et al. 2020; Stevens and Ruxton 2019). Thereby, disruptive colouration and background matching might be expected to involve a different set of behaviours: while background matching is most efficient when accompanied by reduced movement (Ioannou and Krause 2009; Rudh and Qvarnström 2013), disruptive colour patterns are also effective during flight (Creer 2005). Furthermore, disruptive colouration provides camouflage in a wider range of habitats compared to background matching, which allows for higher mobility and wider exploration ranges (Phillips et al. 2017; Stevens et al. 2006). Contrarily, animals displaying background matching might be more restricted to specific micro‐habitats, as background resemblance is an important aspect of their camouflage strategy (Cuthill et al. 2005; Merilaita and Lind 2005). Interestingly, different camouflage strategies have also been shown to contribute to speciation through divergent ecological specialisation (Fulgione et al. 2019; Marques et al. 2017; Nosil et al. 2018).

To understand how camouflage‐linked behaviours could evolve, we have to consider the initiation and neural processing of such behaviours, which in vertebrates involves a coordinated interplay among several brain regions (reviewed here (Branco and Redgrave 2020). Initially, the optic tectum (OT) processes visual information provided by the retinal ganglion cells and integrates auditory and tactile inputs from the brainstem (Westby et al. 1990; Wu et al. 2005). Neurons within the OT transmit this information to the brainstem and spinal cord, initiating movements crucial for innate escape responses (May 2006). While the OT is particularly important for near or looming threats, additional brain regions are involved in the processing of distant or ambiguous threats (Branco and Redgrave 2020). In the diencephalon (DIENC), the hypothalamus modulates active defensive behaviours (Hahn et al. 2019) and initiates fast active‐avoidance behaviours, particularly in larval fish (Lovett‐Barron et al. 2020). Lastly, the telencephalon (TEL), notably the amygdala, plays a pivotal role in the processing of conditioned defensive responses (Roberts et al. 2016; Tovote et al. 2015). Collectively, the OT, DIENC and TEL form an integrated neural circuit essential for detecting threats and initiating camouflage‐linked behaviours, such as escape responses, based on environmental cues and past experiences. Such neural circuits can be subject to evolutionary change, which is known to drive behavioural differentiation (Barker 2021). In fish, for instance, escape response differences are linked with brain‐wide activity changes and differential gene expression in the brain (Pantoja et al. 2020; Xu et al. 2024). Such changes occurred in zebra fish selection lines after only two generations and may thus be implicated in rapid diversification (Pantoja et al. 2020).

To study the evolutionary link between camouflage, behaviour and brain gene expression, we can turn to one of the most captivating colour palettes in the animal kingdom, which is displayed by coral reef fishes. Among these, the hamlet radiation (*Hypoplectrus* spp.) offers a unique opportunity to compare sympatric species with different colour patterns that are otherwise extremely similar ecologically (Holt et al. 2008; Whiteman et al. 2007), morphologically (Puebla et al. 2018, 2022) and genetically (Hench et al. 2019, 2022). All hamlets are diurnal, active from sunrise to sunset, with daily matings at dawn (Fischer 1980; Puebla et al. 2012). During the day, while foraging and patrolling their territories, hamlets are solitary but occur in close proximity to each other (Fischer 1980; Picq et al. 2019). These observations suggest that hamlets are highly similar in various behavioural aspects, such as activity or sociability (understood here as an individual's preference to associate with conspecifics, (Gartland et al. 2022). The most remarkable difference among hamlets lies in their colour patterns, which play a central role in reproductive isolation through visually based assortative mating (Domeier 1994; Fischer 1980; Puebla et al. 2007), with rare hybrid spawnings (~2%; (Puebla et al. 2007, 2012). The diversity of colour patterns in the hamlets seems to have evolved in part through aggressive mimicry, whereby hamlets mimic the colour patterns and behaviours of nonpredatory fishes to enhance hunting success (Puebla et al. 2007, 2018; Randall and Randall 1960; Robertson 2013; Thresher 1978). Nevertheless, while the aggressive mimicry hypothesis underscores the intricate interplay between colouration and behaviour in the hamlet radiation, it does not seem to hold for all species.

**FIGURE 1:**
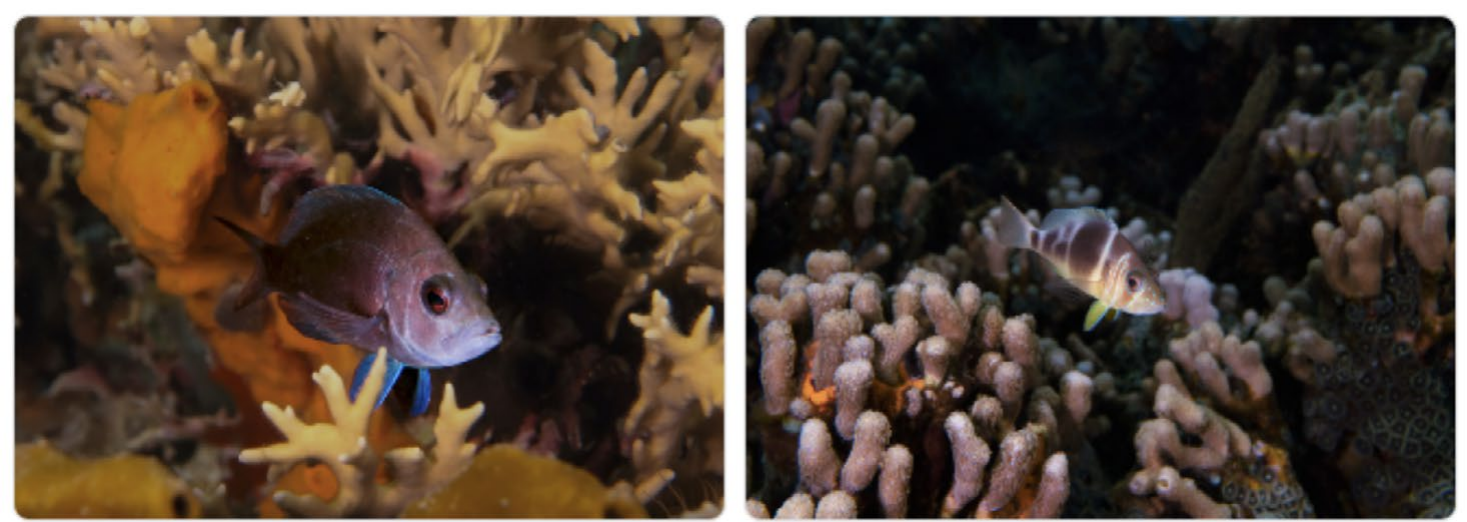
Focal species. A black hamlet (left, *Hypoplectrus nigricans* ) and a barred hamlet (right, *Hypoplectrus puella* ). Individuals were photographed in their natural environment in Bocas del Torro, Panama, where they occur in sympatry. Photographs were taken with flash and post‐edited in Adobe Lightroom by F. Coulmance.

Field observations led us to hypothesise that differences in camouflage‐linked predator avoidance strategies between hamlets may also play an eco‐evolutionary role in their radiation. While the role of camouflage in colour pattern divergence within this genus has previously been discussed (Aguilar‐Perera 2004; Domeier 1994; Lobel 2011; Thresher 1978), it has never been formally tested. In this study, we complemented behavioural assays from the laboratory and the field with brain transcriptomic analyses to compare the predator escape responses of black hamlets ( *Hypoplectrus nigricans* ), characterised by their dark colouration and barred hamlets ( *Hypoplectrus puella* ), brown fish with dark vertical bars that disrupt their body shape (Figure 1). Although hamlets may not represent the most specialised examples of camouflage, their recent radiation provides a unique opportunity to test the evolutionary implications of colour pattern differentiation in morphologically and ecologically almost identical sympatric species. If camouflage is relevant to colour pattern differentiation between black and barred hamlets, we expect that (H1) barred and black hamlets differ in their escape response behaviour, with barred hamlets relying more on flight due to their disruptive colour pattern, while black hamlets would be more inclined to hide in dark recesses. However, (H2) we would not expect differences in nonescape‐related behaviours, such as activity and sociability, due to the species' close relatedness and their similar ecology. Furthermore, (H3) we would expect any escape behaviour differences to be accompanied by transcriptomic differentiation in the brain, particularly in regions responsible for innate rather than learned behaviours (here the OT and DIENC).

## 2 Material and Methods

### 2.1 Sample Acquisition and Ethical Considerations

We collected barred ( *H. puella* ) and black ( *H. nigricans* ) hamlets from reefs around Isla Colón (Bocas del Toro, Panama), choosing the same locations for the behavioural and molecular experiments. Only individuals with unambiguous colour patterns were collected to avoid including potential hybrids. All authors confirm that the welfare of animals was prioritised and respected in the study. We followed the recommended guidelines of the Animal behaviour Society for fieldwork with animals. Fish for the behavioural experiment were handled with great care and released after the completion of the experimental trials approximately four days after capture. A limited number of fish were euthanised for brain transcriptomics following the Institutional Animal Care and Use Committee (IACUC) guidelines. More specifically, individuals were sacrificed by rapid chilling, which does not require any hazardous agents and has been shown to be the most humane veterinary practice for fish (Wilson et al. 2009). Permission for this study was acquired through the Smithsonian Tropical Research Institute ACUC (protocol: SI‐21007) and the Panamanian Ministry of Environment (permit: ARG‐005‐2022).

### 2.2 Laboratory Experiments

In October and November 2022, we collected 50 barred and 47 black hamlets from five reefs around Isla Colón, Panama (Table S1). The experiment was conducted over eight sampling cohorts, each consisting of 10–14 fish with a balanced species ratio per cohort (and a balanced sex ratio since the hamlets are simultaneously hermaphroditic). Fish were captured between 9 AM and 2 PM using hook‐and‐line or hand nets. Postcapture, specimens were transported to the Smithsonian Tropical Research Institute (STRI) Bocas del Toro Research Station, where they were kept in 1x1x0.4 m holding tanks with constant seawater flow under natural light. They received twice‐daily feedings of shrimp meat and were otherwise left undisturbed. Due to space constraints, fish were kept in same‐species and mixed‐species pairs, with each tank containing two hideouts to accommodate both fish comfortably.

Two days after capture, we transferred fish from the holding tank into an experimental tank to individually undergo three sequential behavioural tests: i) activity assessment, ii) escape response test and iii) sociability experiment. These experiments were conducted in a separate room with artificial light, thick walls and a door to prevent noise disturbance. Using PVC pipes, white curtains and a cover, we also removed potentially disturbing visual stimuli. With this, we created two separate spaces, which included a table with one circular white PE tank each (diameter = 79.5 cm, height = 35 cm) without any object inside. Three LED lights were evenly distributed along PVC pipes to standardise light conditions without casting shadows above each arena. It is to be noted that hamlets naturally occur in shallow reefs in sympatry, experiencing similar light regimes. The tank was filled with eight centimetres of water to limit movement to two dimensions principally, which is key for automated video analysis. This water level provided sufficient space for hamlets, with a height of 3–4 cm and a standard length of 6–8.5 cm, to move comfortably. Water was exchanged twice a day and aerated between trials. All experiments were recorded using Akaso Brave 7 LE cameras mounted above the centre of the tank (Figure S1). At the end of the experiment, the fish were returned to their holding tank. The following day, the fish were released at their location of capture.

#### 2.2.1 Activity Assessment

To test for differences in swimming activity between the two species, we ran an open field trial. Therefore, fish were placed in a white opaque plastic tube with a lid (diameter 9 cm) at the centre of the arena. The tube had small holes allowing light to enter, preventing the fish from acclimatising to darkness, which could have caused them to be temporarily blinded when the tube was lifted. We allowed the fish to acclimate in the plastic tube for 3 min, to recover from handling. To start the open field trial, we lifted the container and allowed the fish to move freely in the empty test tank for 10 min (Figure S1A). Using video tracking software, we measured the distance the fish moved in the test tank for 9 min, starting 30 s after release. We waited 30 s because we noted in pilot experiments that after being released, fish sometimes reacted with burst swimming or freezing for a few seconds in reaction to the movements of the experimenter or the curtain.

#### 2.2.2 Escape Response Test

To test for differences in escape response between black and barred hamlets, we performed four simulated predation events. Pilot tests showed that trials could be standardised best when reaching towards the fish by hand rather than, for example, with a model of a predator. The experimenter carefully opened the curtain and moved the right hand towards the fish, until the fish reacted (usually by burst swimming) at which moment the experimenter withdrew the hand from the arena. Escape responses typically lasted for a few seconds only. We repeated the procedure three more times in 15‐s intervals, resulting in 4 trials per fish. From video recordings, we noted: i) the duration of the escape response and ii) the number of quadrants crossed during the escape. The videos allowed us to obtain a highly accurate time estimation of the escape response. Specifically, we measured the time of the burst swimming movement until the fish stopped or transitioned into slow but constant swimming. Rarely occurring second accelerations were not taken into account. We approximated the distance swum during this escape response by virtually dividing the circular tank into four pie‐slice‐like quadrants, which were rotated such that the focal fish was centred in one quadrant at the start of the escape response. Using this approach, we counted the number of quadrants—including the starting one—that the fish swam through during its response. A zero score is possible if the fish did not move at all. Due to the experimenter's hand entering the testing arena, we could not use automated tracking software to extract the exact distance moved during the escape response as we did in the open field trial. In case we could not obtain data for all four repeats, the analysis was based on the number of valid tests obtained for that individual.

#### 2.2.3 Sociability Experiment

We tested the sociability and association preference of hamlet species using a three‐way choice design. Each individual was simultaneously presented with a conspecific, a heterospecific and an inanimate object as a nonsocial control. These con‐ and heterospecific fish originated from a different reef to exclude the possibility that our focal fish has previously encountered either of the two fish presented. The nonsocial control was important to confirm that the approach of the stimulus individuals by the focal individual was an association behaviour rather than just a form of exploratory behaviour. The two stimulus individuals and the inanimate object were positioned in cylindrical clear glass containers (diameter 10 cm), equidistant to each other (Supplementary Figure S1B). We defined an interaction zone of approximately one body length (approximately 7.5 cm) around them. The positions of the stimuli were randomised for each experiment. First, we recaptured the focal individual following the escape response test and placed it back in the white opaque plastic tube in the centre of the test tank. Then, we positioned the two stimulus individuals and the inanimate object in cylindrical clear glass containers and placed them in the experimental tank as previously described. We left the fish undisturbed for a two‐minute acclimation period before lifting the plastic tube and presenting the focal fish with the available choices. Once the tube was lifted, we allowed the focal individual to swim freely in the experimental arena for 10 min and quantified the association time (time inside the interaction zone) towards each stimulus. To avoid any bias related to the position of the stimulus individuals in the arena, we switched their positions after 5 min without an additional acclimation period. We summed the association times in each zone of the two phases (before and after switching) and used this value as our response variable.

### 2.3 Field Study

To validate our findings from the laboratory under natural conditions, we conducted a field study following Nunes et al. (Nunes et al. 2015). Between October 2022 and May 2023, we scored the escape response of 32 black and 62 barred hamlets at 11 reefs around Isla Colón, Panama (Supplementary Table S2). The escape response of each fish was recorded through freediving, where we descended just above the corals to be at the same level as the fish, approximately five meters away from it. From here, we swam horizontally towards the fish at about 1 min per second, extending our right arm towards it. When the fish initiated its escape, we dropped the first marker (a small concrete ring with underwater tape, which we held in our right hand) to mark our position at the moment of escape initiation. The following steps depended on whether the fish (i) escaped or (ii) went into hiding.

For fish that escaped (i), we continued swimming to the exact location from which the fish initiated its escape and dropped a second marker to obtain the fish's position relative to ours at the moment of escape initiation. We then measured the distance between these two markers to obtain the flight initiation distance (FID), a widely used index to assess alertness and decision‐making of prey (Blumstein et al. 2003; Nunes et al. 2015). Upon resurfacing, we recorded the fish's escape reaction (escaping as opposed to hiding) and the FID on underwater paper attached to a buoy that we carried with us. For fish that went into hiding (ii), we retreated backward towards the surface after dropping the first marker. At the moment the fish escaped and hid, we further started a stopwatch and retreated backwards towards the surface after dropping the first marker. As soon as the fish came out of its hideout, we stopped the time and dove back down to measure the FID from the first marker to the point from which the fish went into hiding. The reefs around Isla Colón exhibit characteristic structures such as small corals, rocks or sponges, facilitating the recall of the escape location, especially since the hideout would be in proximity to that specific spot. Fish that initially escaped across the reefs for more than a meter before going into hiding were classified as ‘escape then hide’. After returning to the surface, we recorded the escape response (hiding or escaping then hiding), the FID and the duration of hiding. This freediving experiment was conducted by one person at a time, with a second person in proximity to assist. Trials were conducted at depths ranging from one and six meters between 9:30 AM and 3:30 PM. Hamlets are active and forage throughout the day. Mating displays and spawning occur almost exclusively during the hour preceding sunset, after 5 pm (Fischer 1980). Each fish was scored only once to avoid pseudo‐replication. Therefore, we moved unidirectionally across the reef and refrained from revisiting the same reef, or at least the same area of the reef for large reefs.

### 2.4 Statistical Analyses of Behaviour

Statistical analyses were performed in R v4.3.1 (R Core Team 2023) and results were visualised using ggplot2 v3.4.4 (Wickham 2011). All models were validated and fulfilled their respective model assumptions regarding normality and homoscedasticity of residuals. Model validation information, data tables and scripts can be accessed through GitHub (https://github.com/M‐Heckwolf/hamlet_camouflage_strategies).

#### 2.4.1 Laboratory Experiments—Activity Assessment

The distance swum during the activity assessment was automatically tracked using the software ‘Ethovision XT’ (Noldus Information Technology Company). Tracks were smoothed using the software's ‘track smoothing (lowess)’ option, which is based on 10 samples before and after each sample point. The resulting distances were log transformed and compared using an ANOVA with species as the predictor variable.

#### 2.4.2 Laboratory Experiments—Escape Response Test

Since the escape response videos were assessed by three different people, we first scored the same twelve individuals, which showed a high level of coherence among the three observers (mean response duration: ANOVA: F (2, 33) = 0.18, *p* = 0.834; mean distance swum: GLM (Poisson): Chi‐square = 0.46, *p* = 0.795). Summarising the four repeated trials per individual, we compared the mean individual response duration between species using the lmer() function of the lme4 v1.1–35.1 package (Bates et al. 2015) and species as an explanatory variable and observer as random intercept. The mean response duration was log +1 transformed to meet the model assumption of normal distribution of residuals. The mean distance swum per individual was analysed using the glmer() function of the lme4 v1.1–35.1 package (Bates et al. 2015), with the rounded mean number of quadrants (counts) per individual as the response variable, species as the predictor variable, and observer as random intercept. The data distribution family was set to Poisson. To avoid model singularity, we set the control variable to ‘glmerControl(check.conv.singular =.makeCC(action= “ignore”,tol = 1e‐4)’. Test statistics were extracted using the Anova() function within the car v3.1–2 package (Fox and Weisberg 2019).

#### 2.4.3 Laboratory Experiments—Sociability Experiment

The sociability experiment was quantified by only one person and thus analysed without a random factor using an ANOVA with time in seconds as the response variable, and an interaction of stimulus (rock, barred or black hamlet) and focal individual (black or barred hamlet) as predictor variables. A post hoc test on the significant stimulus effect was run using TukeyHSD() in stats v4.3.1 (R Core Team 2023).

#### 2.4.4 Field Study

The emergence time and FID in the field were analysed using an ANOVA with species as the predictor variable. The escape response type (escape or hide) was analysed using a Chi‐square test for categorical data contrasting the frequencies of escaping or hiding between black and barred hamlets.

### 2.5 Transcriptomics

In June and July 2022, we collected brain samples of six barred and six black hamlets from three reefs around Isla Colón, Panama. After capture, specimens were transported to the STRI Bocas del Toro Research Station, where they were kept in mixed pairs in 1 × 1 × 0.4 m holding tanks with constant seawater flow under natural light until sampling (Table S3). These fish did not undergo any treatment and were not included in the behavioural experiments to avoid stress and ensure transcriptomes reflect their natural differentiation under common garden conditions. Therefore, individuals were kept in the holding tanks for up to 15 days (11 ± 3 days), receiving twice‐daily feedings of shrimp meat, while otherwise being left undisturbed. Due to their sympatric and syntopic occupancy of the same ecological niche (Holt et al. 2008; Puebla et al. 2018; Whiteman et al. 2007)—which we utilised by sampling the same reef/microhabitat within the same time—we expect them to have experienced similar developmental environments, in particular, since colour patterns in these species only start developing approximately three months after hatching (Domeier 1994). Following euthanasia, dissections were carried out in a randomised order between noon and 3 PM to standardise sampling time. Since hamlets spawn in the evening after ~5 PM (Fischer 1980) and do not hydrate new eggs in captivity, the transcription profiles are unlikely to be affected by reproductive behaviours. We dissected and isolated three brain regions (telencephalon, diencephalon and optic tectum), which were individually stored, extracted and sequenced, resulting in three brain samples per individual (*N* = 36).

RNA extraction was performed using the PureLink RNA Mini Kit with an additional TRIzol lysis step (Invitrogen by Thermo Fisher). Sequencing libraries were prepared using the TruSeq Stranded mRNA HT Sample Prep Kit (Illumina), which includes a polyA enrichment step for mRNA purification. All kits were used according to the manufacturer's protocols. Sequencing was conducted on the Illumina NovaSeq platform at the Institute of Clinical Molecular Biology (IKMB), Germany, resulting in 31.1 ± 8.4 (mean ± sd) million 100‐base paired‐end reads per library. Demultiplexed and converted fastq files were quality‐checked using FastQC v0.11.5 (Andrews 2010). Next, we removed Illumina adapters and trimmed low‐quality reads with Trimmomatic v0.36 (Bolger et al. 2014) using a sliding‐window trimming approach. This approach starts at the 5' end and clips the read once the average quality within a four‐base window falls below 25 and only retains reads with a minimum length of 30 bases. After filtering, we retained an average of 24.0 ± 6.5 million high‐quality reads with an average sequence length of 94 ± 1 bases per library, with comparable read numbers for black and barred hamlets (barred = 23.96 ± 6.77 Mio; black = 21.39 ± 5.2 Mio; ANOVA, F 1,34 = 1.629, *p* = 0.21). Filtered reads were aligned against the *H. puella* reference genome (ENA accession: GCA‐900610375) using HISAT2 v2.1.0 (Kim et al. 2015). On average, 11.2 ± 2.9 million reads were uniquely aligned per library (barred = 11.13 ± 3.05 Mio; black = 10.05 ± 2.33 Mio; ANOVA, F 1,34 = 1.427, *p* = 0.241). Since the two species are closely related (*F*_*st*_ < 0.03, Hench et al. 2019), we were able to use the published *H. puella* (barred hamlet) reference genome for both species without introducing any bias (percentage reads uniquely mapped: barred = 46.6 ± 0.8%, black = 47.2 ± 1.6%). Using HTSeq v0.13.5 (Anders et al. 2015), we quantified the number of reads unambiguously mapped per gene.

In R v4.3.1 (R Core Team 2023), we subsetted the data by brain tissue and tested for differential expression using DESeq2 v1.42 (Love et al. 2014). More specifically, we first normalised raw read counts using size factors estimated by the median‐of‐ratios method, which accounts for sequencing depth and RNA composition differences between samples. Next, dispersion estimates were fitted to a parametric model, and the data were analysed using a negative binomial GLM to model count variability. Lastly, differential expression was tested using the Wald statistics, with adjustments for multiple testing made via the Benjamini–Hochberg procedure to control the false discovery rate. A sample clustering approach identified one outlier, probably due to low RNA concentration (21.3 ng/μl compared to the mean ± sd: 151 ± 46 ng/μl), which was removed from subsequent analyses (1 x *H. nigricans* —diencephalon). To obtain a functional overview of differential expression, we conducted a conditional hypergeometric Gene Ontology (GO) term enrichment analysis. Therefore we used our filtered expression counts dataset as the gene universe to compare against our differentially expressed genes using the packages GOstats v2.68 (Falcon and Gentleman 2007), GSEABase v1.64 (Morgan et al. 2023) and goEnrichment v1.0 (Asis Hallab 2015). Figures were plotted using ggplot2 v3.4.4 (Wickham 2011) and pheatmap v1.0.12 (Kolde and Kolde 2018).

## 3 Results

### 3.1 Escape Response Behaviour

**FIGURE 2:**
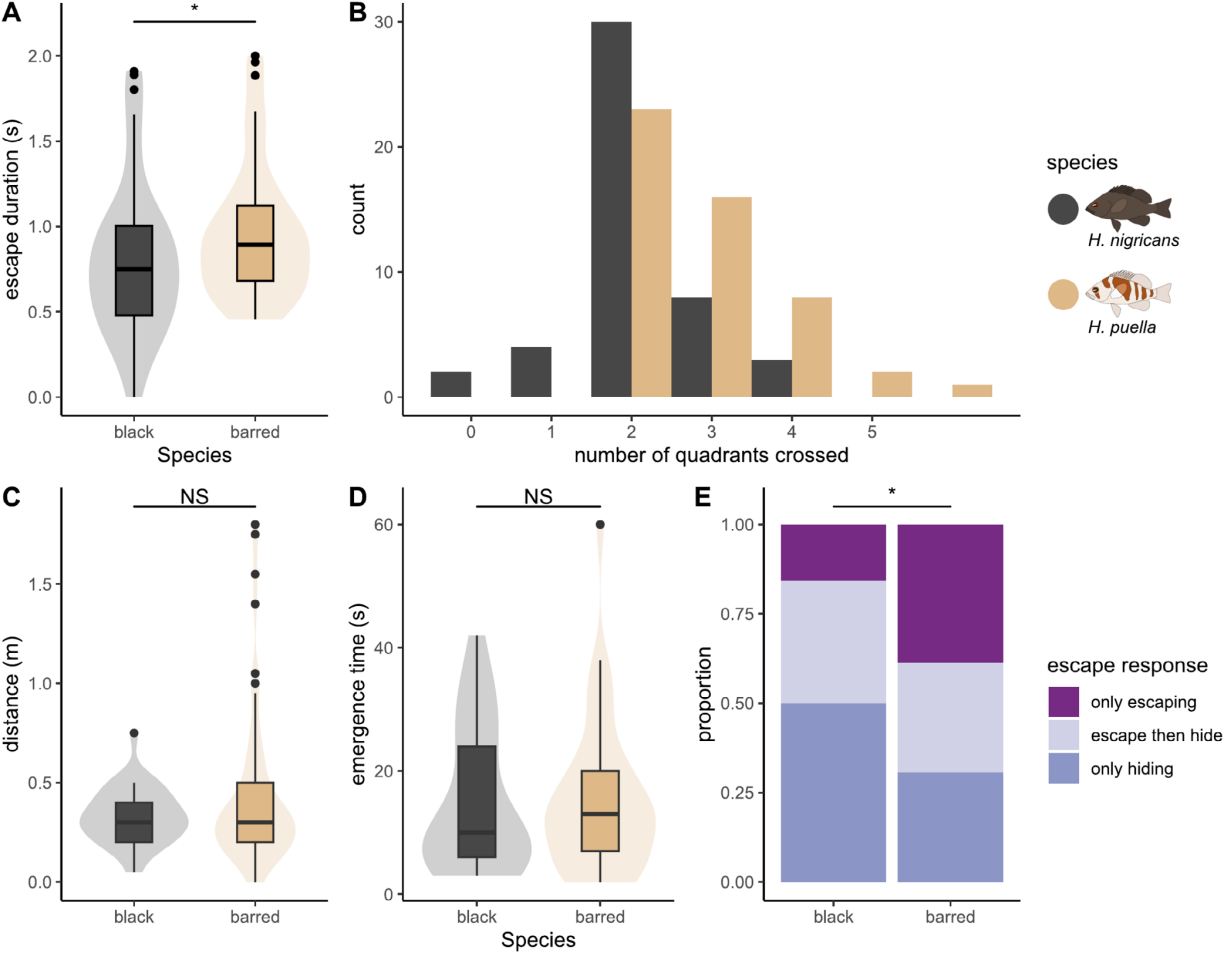
Difference in escape response between black and barred hamlets. Escape response parameters were measured both in the laboratory (A–B) and in the field (C–E). These parameters include: (A) the duration of the escape response in the tank, (B) the distance swum during the escape response in the tank (as count of quadrants the fish swam through; higher numbers indicate greater distance), (C) the flight initiation distance in the field, (D) the time until individuals re‐emerge from hiding in the field (only including the fish that went into hiding) and (E) the type of escape response in the field as a proportion. Boxes in A–D show the median with 25th and 75th percentiles. Whiskers indicate values within 1.5 times the interquartile range, and circles represent values outside this range. Shaded density kernels represent the frequency and distribution of raw data points. Barred hamlets are indicated in brown and black hamlets in dark grey.

To investigate behavioural differences between black and barred hamlets, the escape response was measured in the laboratory and the field. Laboratory experiments on 50 barred and 47 black hamlets showed that the escape response duration was slightly but significantly longer in barred (~0.96 s) compared to black hamlets (~0.80s) (Figure 2A; LMM: Chi‐squared (1) = 5.73, *p* = 0.017). Correspondingly, barred hamlets swam on average across three quadrants during their escape, which is significantly farther than the two quadrants on average in black hamlets (Figure 2B; GLMM: Chi‐squared (1) = 4.89, *p* = 0.027). To validate the differences in escape responses in the hamlets' natural environment with potential hideouts, we conducted a field survey encompassing 94 individuals (62 barred and 32 black hamlets). While the flight initiation distance (Figure 2C; F (1,92) = 1.98, *p* = 0.163) and the time until re‐emergence (Figure 2D; F (1,92) = 0, *p* = 0.999) did not differ between species, we observed a difference in the type of escape response, with barred hamlets utilising multiple strategies, while black hamlets predominantly went into hiding or escaped and then hid, and very rarely only escaped (Figure 2E; Chi‐squared (1) = 4.61, *p* = 0.032 hiding vs. escape; Chi‐squared (2) = 5.86, *p* = 0.053 all three groups).

### 3.2 Nonescape Behaviours

To show that behavioural differentiation is not a general phenomenon between the species but occurs specifically in camouflage‐related behaviours, we also tested two nonescape behaviours: activity and sociability. As expected, we did not find any differences in activity between the two species, here measured as the overall distance swum and the swimming velocity during the activity assessment (Supporting Information Figure S2). Furthermore, they also did not show any differences in sociability or association preference between con‐ and heterospecifics (Supporting Information Figure S3), which we tested outside of the mating context. Both barred and black hamlets showed a preference for associating with another fish, regardless of whether it was conspecific or heterospecific, rather than with an inanimate control object (ANOVA: F (2,285) = 64.87, *p* < 0.001; TukeyHSD, *p* < 0.001).

### 3.3 Brain Transcriptomics

**FIGURE 3:**
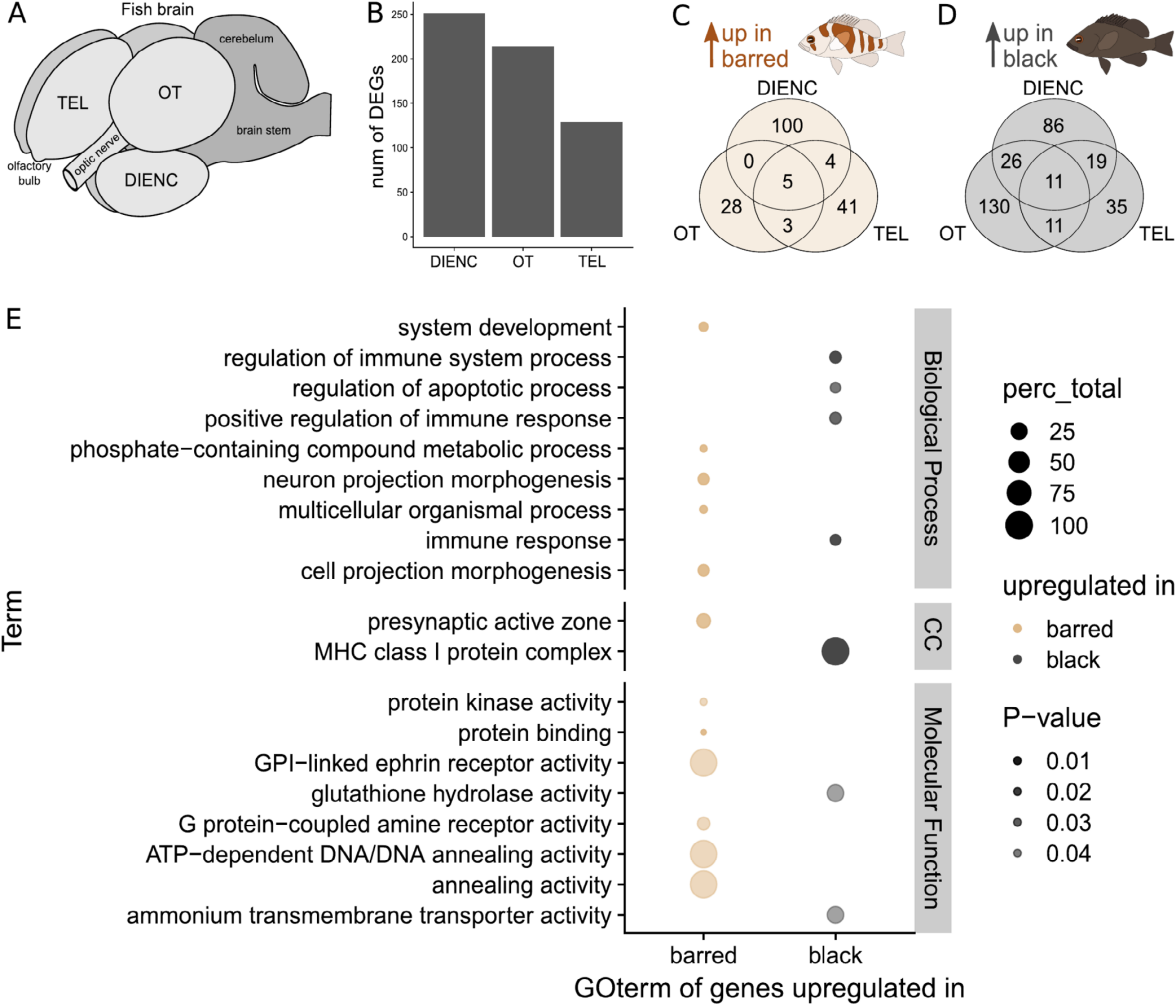
Brain transcriptomics. (A) Schematic overview of a fish brain indicating the regions used for brain transcriptomics: The optic tectum (OT), telencephalon (TEL) and diencephalon (DIENC). (B) Bar graph showing the number of DEGs per brain region. Venn diagrams of differentially expressed genes within the three brain regions that are upregulated in barred (C) and black (D) hamlets. (E) GOterm enrichment analysis results. Top 9% of the terms with lowest adjusted *p*‐values for all genes upregulated in barred or black hamlets across biological processes, cellular components (CC) and molecular functions. The number of DEGs compared to all genes with each specific GOterm is indicated as percentage of total in the circle size, while adjusted *p*‐values are visualised as colour transparency.

We dissected and sequenced the transcriptomes of three brain regions, the optic tectum (OT), the diencephalon (DIENC) and the telencephalon (TEL), in six barred and six black hamlets (Figure 3A). From these 36 samples, we obtained 11.2 ± 2.9 (mean ± sd) million filtered high‐quality reads that were uniquely aligned and analysed for differential expression. One outlier sample was dropped during the analysis (1 x *H. nigricans* —diencephalon). Out of the three brain regions, the highest number of differentially expressed genes (DEGs) was found in the DIENC (251), followed by the OT (214) and the lowest number of DEGs was found in the TEL (129) (Figure 3B, Supporting Information Figure S4). In the OT, most DEGs were downregulated in barred hamlets compared to black hamlets (36 up in barred, 178 up in black), while the numbers were more even in the other two brain regions (TEL: 53 up in barred, 76 up in black; DIENC: 109 up in barred, 142 up in black; Figure 3C,D, Supporting Information Table S4).

To understand the functional implications of these expression differences, we tested for enriched gene ontology terms combining the DEGs across brain regions for more robustness but separating genes upregulated in black or barred hamlets (Figure 3E). We found the DEGs enriched for immune and oxygen‐related processes to be upregulated in black hamlets in the OT, while the DIENC contained the neuronal development and signalling genes upregulated in barred hamlets (Figure 3E). Additionally, black hamlets showed enrichment for cell development and barred hamlets for processes associated with neuron organisation and synaptic activity, suggesting distinct functional specialisations between the species.

Focussing on individual genes, we found five and 11 genes that were consistently upregulated in barred and black hamlets across all three brain regions, respectively (Supporting Information Table S5, Figure 3C,D). In black hamlets, two haemoglobin subunits involved in oxygen transport were consistently upregulated across all brain regions. Furthermore, CLDN20, which is involved in the blood–brain barrier, and NLRP12, a negative regulator of inflammatory responses, were consistently upregulated in black hamlets. For genes upregulated in barred hamlets, we found biological processes related to neuron and cell development to be enriched. In line with this, a gene consistently upregulated in barred hamlets, PCDHA7, guides the establishment and maintenance of specific neuronal connections in the brain. Another interesting gene upregulated in barred hamlets is DNASE1L1, which plays a role in denucleation to prevent clouding of the eye lens (Zhang et al. 2020). On the other hand, DNASE1L3, which is upregulated in black hamlets, mediates the denucleation process in the blood to generate cell‐free DNA in blood circulation (Watanabe et al. 2019). However, their specific roles in the brain are not well established.

**FIGURE 4:**
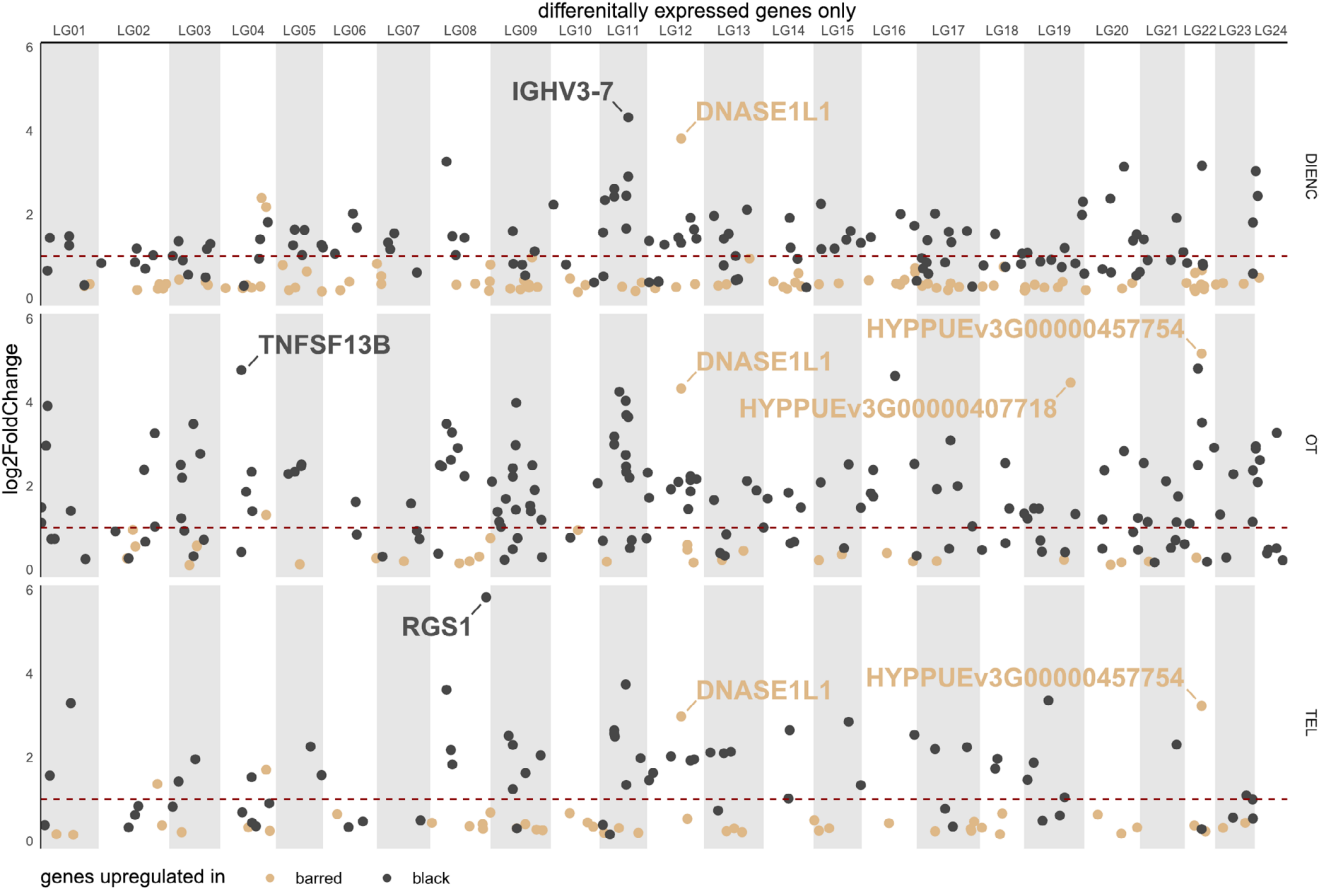
Differentially expressed genes plotted as log2 fold change values along the genome for each of the three brain regions (DIENC, OT, TEL). A dashed line at one indicates a doubling in expression and colours visualise if genes were upregulated in barred (brown) or black (grey) hamlets. All genes and their adjusted *p*‐values are plotted in Supplementary Figure S5.

When characterising the spatial context of the DEGs, we see that they were not concentrated in specific genomic regions—like the genomic peaks of differentiation—but rather spread out along the genome (Figure 4). It is interesting to note that we did not only find more genes upregulated in black hamlets, but also the magnitude (log2 fold change, L2FC) of upregulation was much higher in black compared to barred hamlets (Figure 4). Among the genes with the highest L2FC value in black hamlets were RGS1—which inhibits signal transduction by inactivating the G protein alpha subunits, TNFSF13B—shown to play an important role in the proliferation and differentiation of B lymphocytes, and IGHV3‐7—an immunoglobulin region that participates in the antigen recognition. Among the genes with a particularly high L2FC in barred hamlets were DNASE1L1 and two novel genes, HYPPUEv3G00000407718 and HYPPUEv3G00000457754, which seem to have catalytic properties and transferase activity.

## 4 Discussion

### 4.1 Behavioural Divergence

If hamlets were utilising different camouflage strategies, we would expect barred hamlets to rely more often on flight, due to their disruptive colour pattern, while black hamlets would rely on hiding to achieve background matching in dark hideouts. In line with this hypothesis, barred hamlets showed an increased duration and distance of their flight response in a controlled experiment. In the field, black hamlets showed a preference to go into hiding (> 80%), while barred hamlets—despite exploiting both strategies—more often escaped. Since predator avoidance behaviours are so fundamental to survival, they are often considered hard‐wired and species‐specific, which means that they would have the potential to implicate evolutionary trajectories. However, behavioural variation depending on the structural complexity of the habitat has been observed (Nunes et al. 2015; Yilmaz and Meister 2013). Since black and barred hamlets are highly sympatric, they are exposed to the same predators, light conditions and reef structures to choose hideouts from. Moreover, the observed escape behaviour differences between species were consistent across reefs, suggesting that they might at least in part be heritable. Evidence that such behaviours may be implicated in rapid diversification comes from zebrafish larvae selection lines, which showed differential brain‐wide activity linked to escape response differences after only two generations (Pantoja et al. 2020). However, we cannot exclude that learning has altered their responses, as it has been shown that experience can alter the decision‐making process, for instance through learned suppression of escape (Lenzi et al. 2022).

Strikingly, the behavioural differences between the species did not extend to nonescape‐related behaviours, suggesting that differential predator escape strategies play a role in their divergence. For instance, we found similar levels of activity and similar but low levels of sociability during the day, which were to be expected since hamlets are diurnally active fish that live solitarily, forage for food across the reef and show a varying extent of territoriality (Fischer 1980; Picq et al. 2019). They only loosely interact with con‐ and heterospecific hamlets throughout the day, but gather in conspecific pairs during sunset to spawn (~2% hybrid spawning; (Puebla et al. 2007, 2012), a time window that we specifically excluded for our assessments. While we were only able to test a limited number of nonescape‐related behaviours, our results are in line with previous studies that failed to show any ecological differences between the two species, highlighting their low level of divergence and high ecological similarity (Hench et al. 2022; Holt et al. 2008; Puebla et al. 2018, 2022; Whiteman et al. 2007). Interestingly, nonmating‐associated behavioural differences within and between species have been shown before (Picq et al. 2019; Puebla et al. 2007, 2018; Randall and Randall 1960; Robertson 2013; Thresher 1978). The most prominent behavioural differences that have been recorded to date are linked to the aggressive mimicry behaviour of the hamlets and thus also heavily implicate colour pattern evolution.

### 4.2 Linking Behavioural Differences With Brain Transcriptomics

Since behaviour and brain transcriptomic profiles are tightly associated in fish (Vu et al. 2020), we investigated this link for the escape response in barred and black hamlets. Our transcriptomic study focussed on three brain regions involved in the perception of predators and the initiation of escape responses: the optic tectum (OT), telencephalon (TEL) and diencephalon (DIENC) (Figure 3A). We anticipated substantial differences in gene expression between the species, particularly in regions responsible for innate rather than learned behaviours. In line with this expectation, we found the number of DEGs to be higher in OT and DIENC—which are important to detect looming and ambiguous threats—compared to TEL—which plays a stronger role in conditioned behaviours (Branco and Redgrave 2020). This finding may again suggest that the escape response differences are evolved rather than learned. Since the TEL includes large parts of the mesolimbic reward system and the social decision‐making axis (Soares 2017), the lower number of DEGs was further very much in line with the absence of behavioural differences in our sociability experiment.

Functional analyses revealed that gene upregulation in the DIENC of barred hamlets was related to neuron organisation, development and synaptic signalling, suggesting enhanced neuronal plasticity and potentially synaptic activity. Since barred hamlets were more dynamic in their escape response than black hamlets, the high neuronal plasticity could provide a means by which they quickly adjust their escape strategy. Furthermore, the DIENC acts as a sensory relay station (Butler 2008), which might indicate that the species processes environmental information differently. This would be in line with the fact that disruptive colouration provides camouflage in a wide range of habitats allowing for greater exploration ranges (Phillips et al. 2017; Stevens et al. 2006), which could result in a higher sensory input and thus require a more dynamic neuronal and synaptic signalling. While we did not test for exploration differences between these hamlets, this might be an exciting direction for future research.

Since gene expression is energetically costly, it not only involves trade‐offs between expressed genes but also with other metabolic functions (Weiße et al. 2015). Thus, it is interesting to see the high L2FC for genes upregulated in black hamlets (Figure 4), suggesting that they invest much more into the expression of these genes than barred hamlets do. However, such comparisons are relative and do not relate to an absolute higher expression across all genes in black hamlets (e.g., Supplementary Figure S6). While RNA sequencing can reveal proportional changes in gene expression, the total number of reads sequenced—which was equal between the species—depends on technical decisions and does not reflect the overall investment into gene expression per individual (Supporting Information Figure S7). At this point, it is important to mention that we carefully considered relevant quality metrics, normalisation steps and mapping rate (e.g., Supporting Information Figures S6–S8), which do not show any normalisation or mapping bias. Thus, we are convinced that this result reflects a real biological difference rather than a technical artefact. Such a broad upregulation, particularly of immune and oxygen‐related genes, could hint towards a general stress response (Aluru and Vijayan 2009). However, we did not find any evidence for that as none of the typical stress response genes implicated in cortisol release, such as CRHR1, NR3C1, or NR3C2, were differentially expressed between the species (Pagliaccio et al. 2014; Velders et al. 2011). Investing proportionally more into the expression of some genes, as black hamlets do, means that they have either less energy left for other processes, or they have an overall lower expression in other genes, which is potentially more subtle and thus not captured by our DEG analysis. The enrichment of genes mediating neuronal development and morphogenesis in barred hamlets could hint towards a trade‐off between gene expression and cell proliferation and development between the species. While we cannot derive an answer to this from our data, future research could test the RNA/DNA ratio in brain cells or compare brain morphology using micro‐CT scans to anticipate the energy allocation prioritisation between the species.

### 4.3 Eco‐Evolutionary Consequences for the Hamlet Radiation

Although the hamlet radiation is largely phylogenetically unresolved (Hench et al. 2022), the black and barred hamlets from Bocas del Toro used within this study form distinct phenotypic and genetic clusters (Coulmance et al. 2024; Hench et al. 2019). In contrast to a few sharp peaks of genomic differentiation in an otherwise lowly differentiated genome, the spatial distribution of DEGs across the genome indicates a widespread impact of species differentiation on gene expression. Since the regions of genomic differentiation harbour multiple transcription factors (Hench et al. 2022), the widespread gene expression divergence could—at least in part—be the downstream result of differentiation within key elements of their gene regulatory networks. Only a few of these DEGs showed consistent expression differences across all brain tissues, raising the possibility that their differential expression in the brain is simply a by‐product of evolved species‐specific expression patterns that potentially serve a functional role outside of the brain. However, the majority of DEGs were unique to specific brain regions, implying that their differential expression is functionally relevant within those tissues.

Previously, aggressive mimicry, where a predatory hamlet mimics a nonpredatory fish to enhance hunting success, has been suggested as a driver of colour divergence in hamlets (Puebla et al. 2007, 2018; Randall and Randall 1960; Robertson 2013; Thresher 1978). Interestingly, their aggressive mimicry does not only involve the colour pattern similarity to their model but also a change in the hamlets' behaviour, such as body positioning and swimming patterns (Puebla et al. 2007; Randall and Randall 1960), providing an example of the intricate link between behaviour and colour patterns in the hamlets. Noteworthy, while the aggressive mimicry hypothesis is well established for some hamlet species, it has not been proven for others, such as the two species we investigated here, leaving room for alternative explanations. Our results, suggesting divergent camouflage strategies between black and barred hamlets, add another example of an association between colouration and behaviour outside the mimicry context. If colouration and potentially hard‐wired escape behaviours were to co‐evolve, a mismatch between them in hybrids might reduce their fitness and act as a postzygotic barrier. However, while predator escape behaviours are often assumed to be hard‐wired (Yilmaz and Meister 2013), individuals can adjust this behaviour to some extent (Lenzi et al. 2022). But even without a distorted link between colouration and behaviour, hybrids showing intermediate colour patterns (Domeier 1994) may not be optimally camouflaged in their environment and could be subjected to higher predation rates. However, assessing the predator perspective was beyond the scope of our study.

The evolution of colour patterns for camouflage has previously been discussed in hamlets. For example, Lobel (2011) speculated that the blue coloured maya hamlet (*H. maya*) evolved camouflage colouration towards a bright blue tunicate, *Clavelina puerto‐secensis* Also barred and indigo hamlets ( *H. indigo* , blue with vertical bars) have previously been mentioned for their cryptic colouration (Domeier 1994; Thresher 1978). However, for black hamlets, the opinions seem to diverge with Domeier (1994) describing blacks as having ‘concealing colouration’, while Thresher (1978) mentioned that black hamlets ‘stand out on the reef’. It is important to note that black hamlets vary greatly in morphology and colouration across the Caribbean, potentially due to ongoing speciation (Aguilar‐Perera 2004). In line with this, black hamlets have been observed to show different swimming behaviours with individuals from Roatan sticking close to the bottom, while black coloured hamlets from the USVI and lesser Antilles can be found out in the open and even over sand (Robertson DR, personal communication). One explanation for this could be that the link between behavioural strategies and colour morph is environment‐dependent, as it is for instance the case in cichlids (Lehtonen et al. 2023). Whether the differences in swimming behaviour among black hamlets translate into predator escape response differences is unclear, but they seem to go hand in hand with significant size differences with black hamlets from Roatan being considerably smaller (Robertson DR, personal communication). Indeed, size has been shown to modulate predator escape responses, with smaller individuals showing an earlier flight response (Polverino et al. 2024). However, the black and barred hamlets from Bocas we used within this study did not differ in size and are representative of the size range of these populations (ANOVA; F 1,95 = 0.118; *p* = 0.732; mean SDL (cm): blacks: 7.34, barred: 7.30). Interestingly, the black hamlets from Bocas are genetically diverged from other black hamlets across the Caribbean and other hamlet species in general (Hench et al. 2022). Given their spatial, phenotypic, behavioural and genetic variation, it remains to be tested whether our observations represent a local exception or a global phenomenon in the hamlets.

## 5 Conclusion

Our study uncovered consistent differences in predator escape responses and brain transcriptomic profiles between black and barred hamlets, suggesting divergent camouflage strategies between the species. Barred hamlets, with their more dynamic escape responses, show enhanced neuronal plasticity in the diencephalon, suggesting an increased capacity to rapidly adjust to varying threats. On the other hand, black hamlets, which tend to hide from predators, invested more in immune regulation and oxygen transport in the optic tectum, the primary brain region to detect looming threats. The fact that two brain regions crucial for perceiving and initiating escape responses showed higher levels of differentiation compared to the region processing conditioned behaviour hints towards an evolved rather than a learned difference in escape response. Such evolved differences in camouflage strategies would ultimately subject the two species to divergent selective pressures, reinforcing their differentiation despite their sympatric occurrence, particularly if hybrids possess suboptimal camouflage due to intermediate colour patterns and a mismatch in escape behaviour. Our findings suggest that divergent camouflage strategies could be associated with colour pattern evolution in the hamlets, at least for some species pairs or populations. Thus, we provide a complementary explanation to the aggressive mimicry hypothesis, with both drivers heavily relying on an intricate link between colour pattern evolution and behaviour.

## Author Contributions

M.J.H., J.G., M.G.‐S., J.F., W.O.M. and O.P. were involved in different aspects of the conceptualisation and methodology of the study. M.J.H. and J.F. were responsible for the data curation. The formal analysis and investigation was conducted by M.J.H., J.G. and M.G.‐S., with F.C. involved in the investigation. M.J.H., J.G., W.O.M. and O.P. have acquired funding for this project, with M.J.H., W.O.M., and O.P. being responsible for project administration. Resources and supervision were provided by W.O.M. and O.P., with the provision of software by J.G. M.J.H visualised the results and wrote the original draft together with J.G. All authors reviewed and edited the draft version and approved the final manuscript version.

## Conflicts of Interest

The authors declare no conflicts of interest.

## Benefit‐Sharing Statement

A research collaboration was developed with scientists from an institution within Panama (here the Smithsonian Tropical Research Institute), which is the country from which the genetic samples originated. All collaborators are included as co‐authors. More broadly, our group is committed to international scientific partnerships, as well as institutional capacity building. This research follows the Nagoya Protocol on Access and Benefit‐sharing and obtained a certificate of compliance from the Access and Benefit‐sharing Clearing House (ABSCH‐IRCC‐PA‐262087‐1).

## Supporting information


Data S1.

**Table S1.** Laboratory behavioural experiment. Meta data and parameters measured in the laboratory during the activity assessment, the escape response test and the sociability experiment.

**Table S2.** Field behavioural experiment. Meta data and parameters measured in the field during the escape response experiment.

**Table S3.** Brain transcriptomic analysis. Meta data, RNA extraction and read mapping parameters for the brain transcriptomic experiment.

**Table S4.** Brain transcriptomic results. Test statistics and description of differentially expressed genes.

**Table S5.** Highlighted brain transcriptomic results. A list of genes consistently differentially expressed across all three brain regions. For a list of all DEGs see Table S4.

## Data Availability

Raw RNA sequence reads are uploaded to ENA (project accession number PRJEB76552) and will become publicly available before publication. Metadata for all Individuals sequenced as well as individuals' responses within the behavioural assay are attached as supplementary tables (but can alternatively also be uploaded to e.g., Dryad before publication).
