# Supplementary material for: Differences in Colour Pattern, Behaviour and Gene Expression in the Brain Suggest Divergent Camouflage Strategies in Sympatric Reef Fish Species
Source: Mol Ecol. 2025 Apr 29;34(11):e17748. doi: 10.1111/mec.17748 (PMC12100586; doi:10.1111/mec.17748)
Supplement: Supplementary file 1 — Data S1. [file MEC-34-e17748-s006.docx]

**Supplementary Figures**

**A**

**
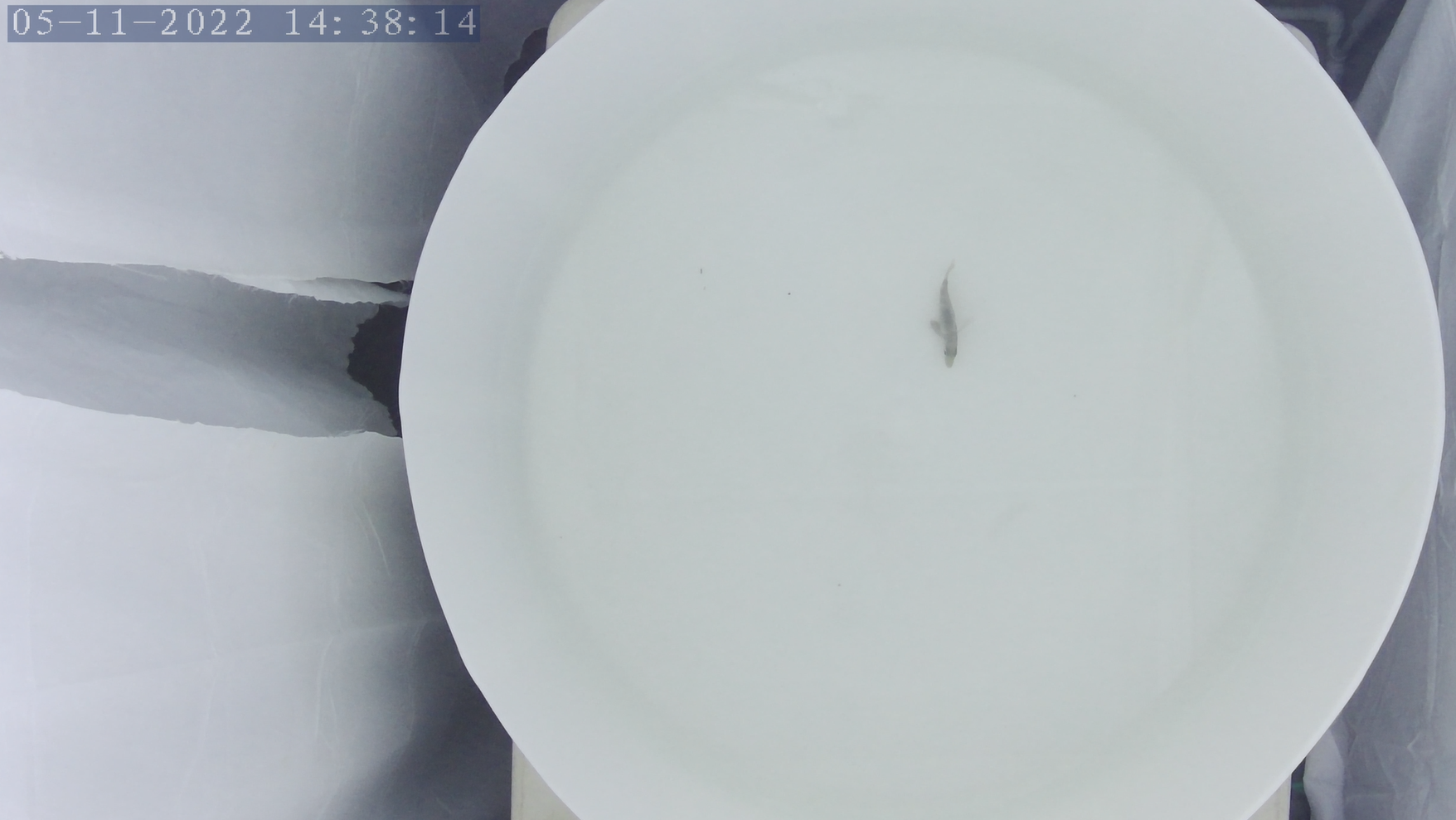
**

**B**

**
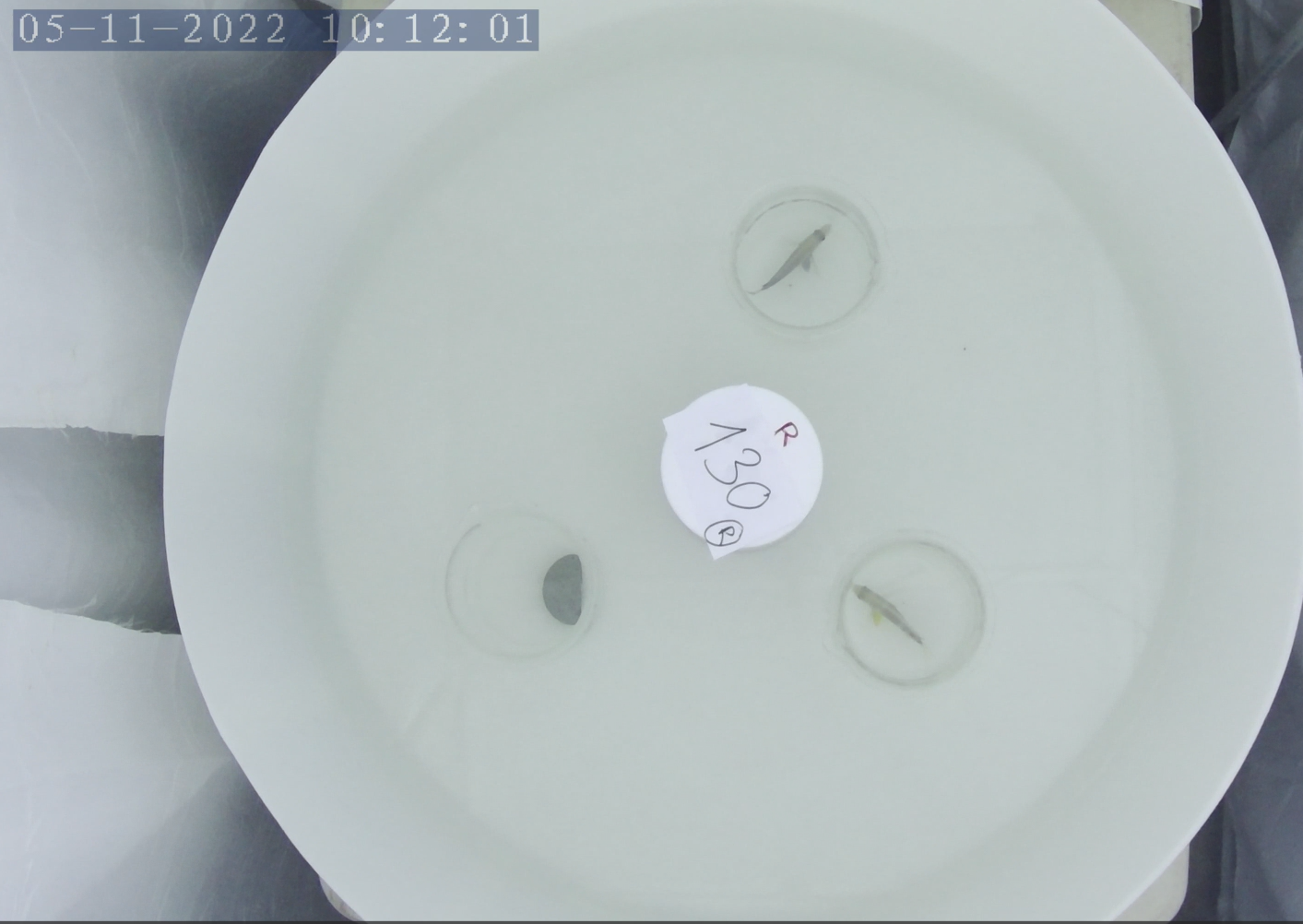
**

**Supplementary Figure S1**: **Setup for the laboratory experiment.** (A) Activity assessment in an open field trial. The focal fish was left undisturbed in an empty tank for ten minutes while its activity was tracked. (B) Sociability experiment. The focal individual was placed in the tube in the centre and presented with three stimuli distributed at equal distances from the focal individual in the tank. The starting positions of each stimulus were assigned randomly, but the two stimulus individuals were switched after five minutes, which was equivalent to half of the experimental time.

**
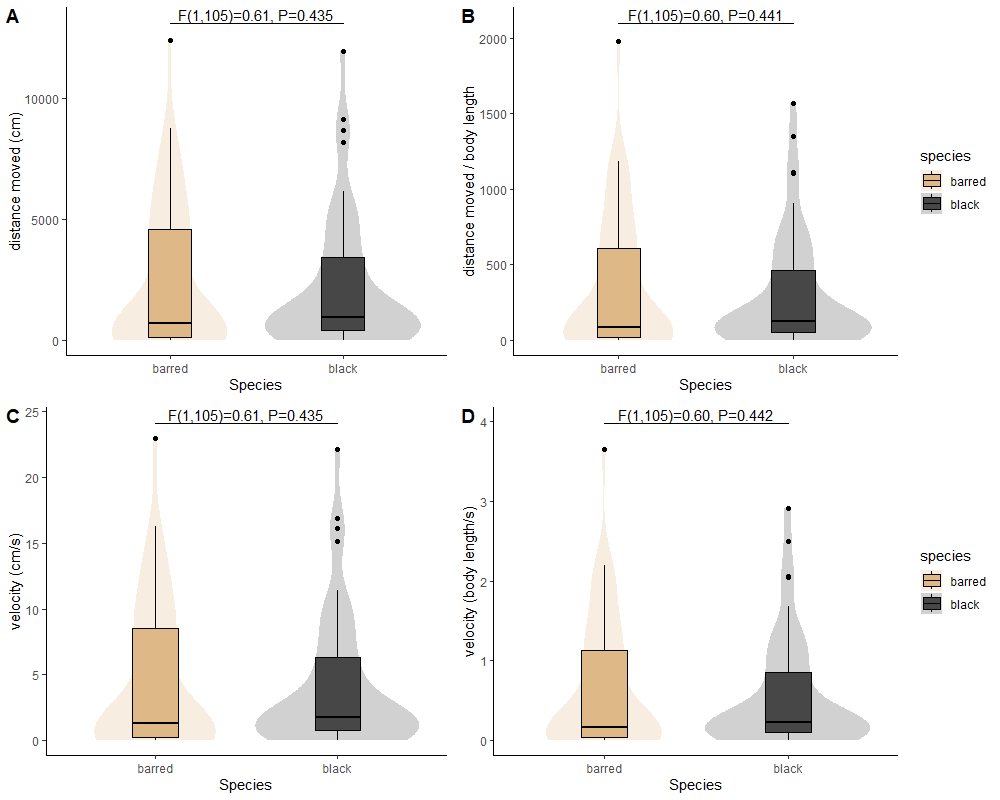
**

**Supplementary Figure S2:** **Activity assessment.** Distance and velocity measurements recorded during a 10 minute open field trial: (A) Distance moved in centimetres, (B) distance moved per body length, (C) velocity in centimetres per second, and (D) velocity in body length per second. Boxes in A-D show the median with 25th and 75th percentiles. Whiskers indicate values within 1.5 times the interquartile range, and circles represent values outside this range. Shaded density kernels represent the frequency and distribution of raw data points. An ANOVA on log-transformed response variables did not reveal differences between black and barred hamlets’ swimming behaviour during the open field trial. F-test statistics can be found in the plot.

**
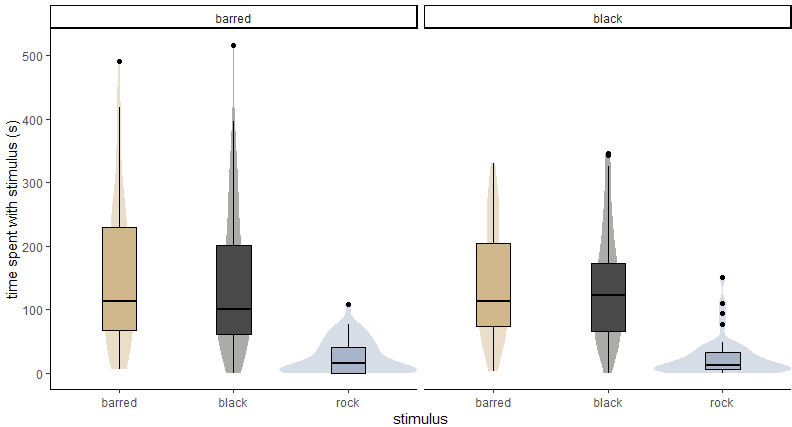
**

**Supplementary Figure S3: Sociability experiment.** The time spent in seconds with either stimulus (barred hamlet, black hamlet or rock) is displayed separately for the two focal species. Boxes show the median with 25th and 75th percentiles. Whiskers indicate values within 1.5 times the interquartile range, and circles represent values outside this range. Shaded density kernels represent the frequency and distribution of raw data points. The experimental duration was 10 minutes. Focal fish were free to explore the rest of the tank and spend time not associating with either stimulus, resulting in association times between 0 and 600 seconds (=10 minutes). We found no difference in association time between the two species (ANOVA: F (1,285) = 0.60, P = 0.440), nor was there a significant interaction effect between the focal species and the stimulus (ANOVA: F (2,285) = 0.18, P = 0.834). However, both barred and black hamlets showed a preference for associating with another fish, regardless of whether it was conspecific or heterospecific, rather than with the rock (ANOVA: F (2,285) = 64.87, P < 0.001; TukeyHSD, P < 0.001).

**
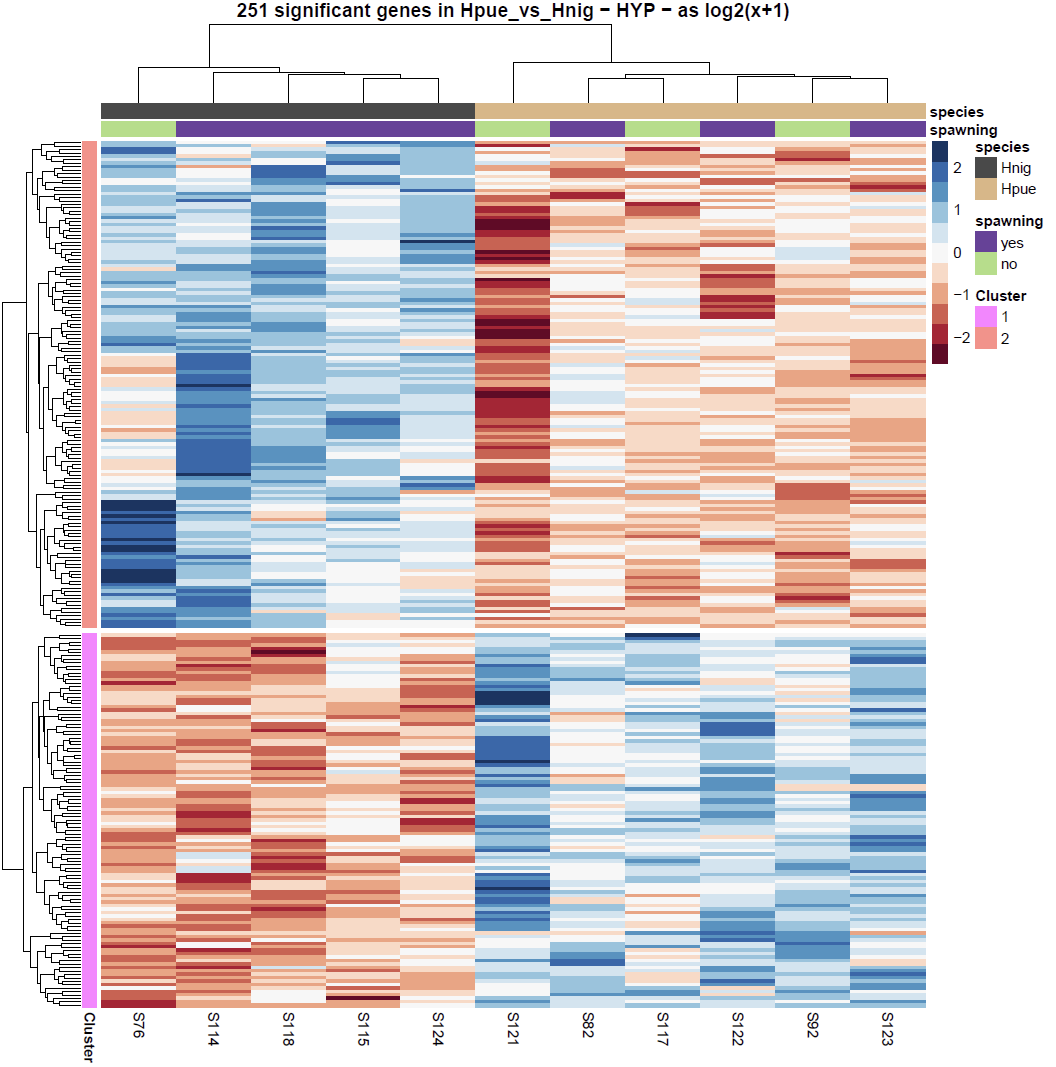
**

**Supplementary Figure S4A:** Heatmap of 251 differentially expressed genes in the diencephalon. Samples are grouped together through unsupervised clustering along the x axis and colours indicate the species name (Hnig = *H. nigricans*, Hpue = *H. puella*). Genes were allowed to cluster in two groups which are equivalent to the genes upregulated in black hamlets (Hnig, cluster 1) and those upregulated in barred hamlets (Hpue, cluster 2).

**
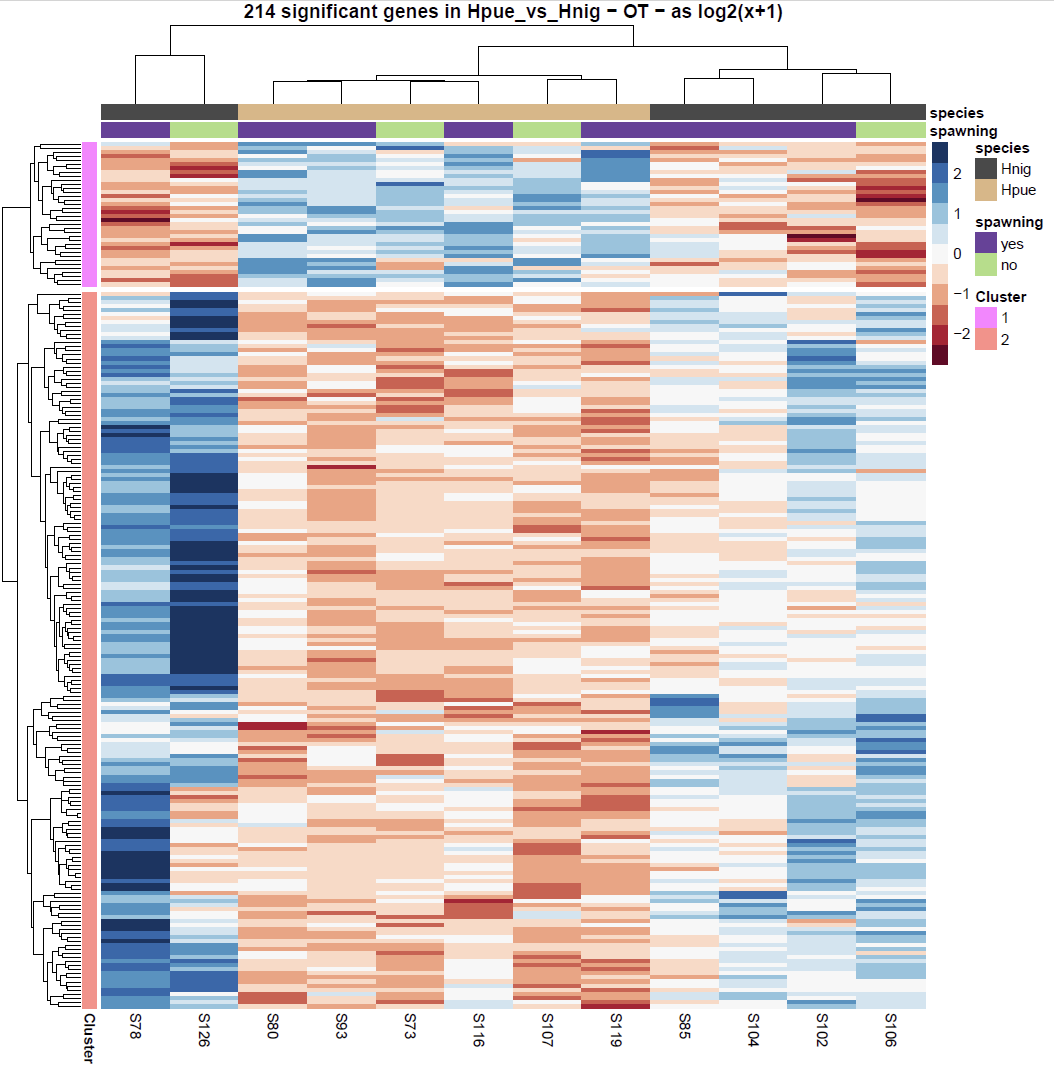
**

**Supplementary Figure S4B:** Heatmap of 214 differentially expressed genes in the optic tectum. Samples are grouped together through unsupervised clustering along the x axis and colours indicate the species name (Hnig = *H. nigricans*, Hpue = *H. puella*). Genes were allowed to cluster in two groups which are equivalent to the genes upregulated in black hamlets (Hnig, cluster 1) and those upregulated in barred hamlets (Hpue, cluster 2).

**
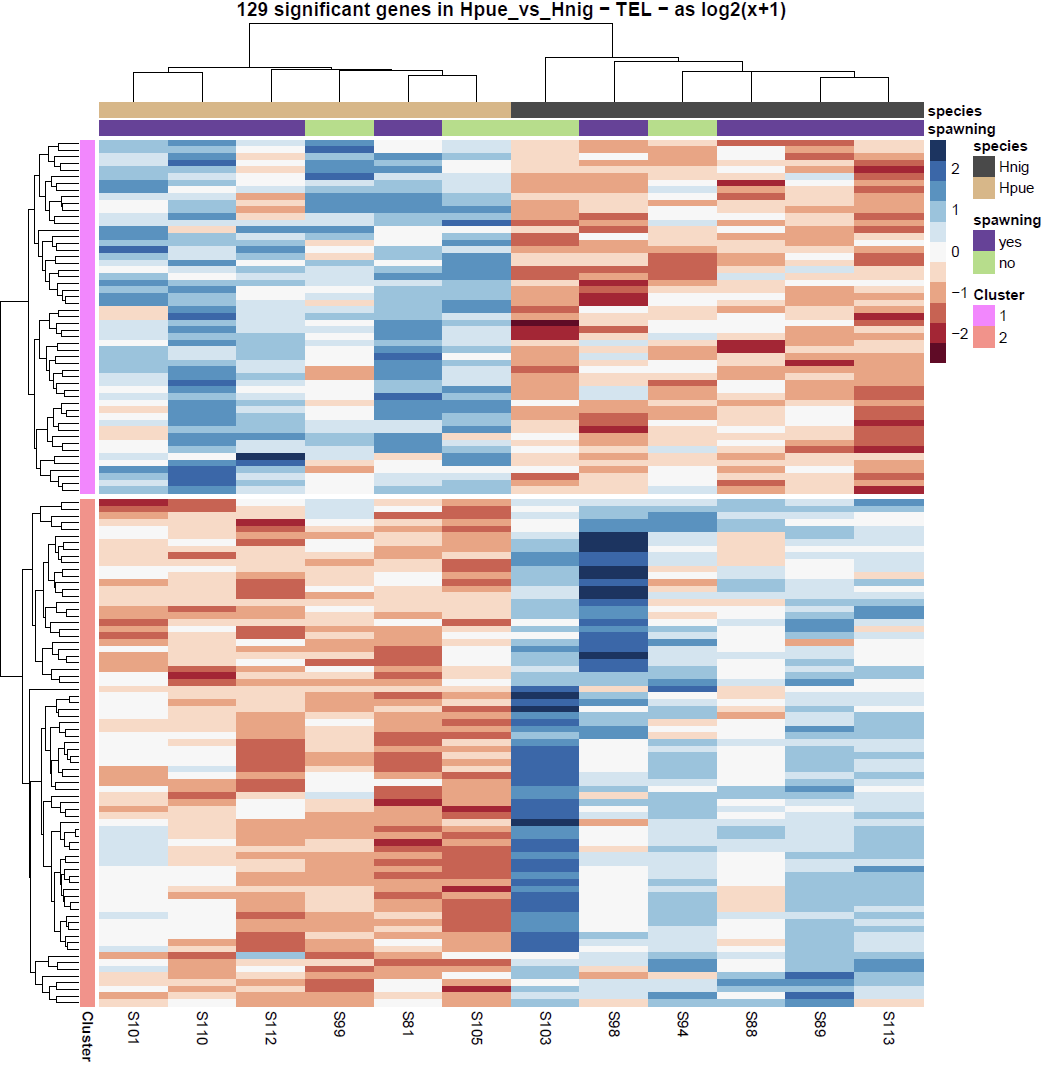
**

**Supplementary Figure S4C:** Heatmap of 129 differentially expressed genes in the telencephalon. Samples are grouped together through unsupervised clustering along the x axis and colours indicate the species name (Hnig = *H. nigricans*, Hpue = *H. puella*). Genes were allowed to cluster in two groups which are equivalent to the genes upregulated in black hamlets (Hnig, cluster 1) and those upregulated in barred hamlets (Hpue, cluster 2).


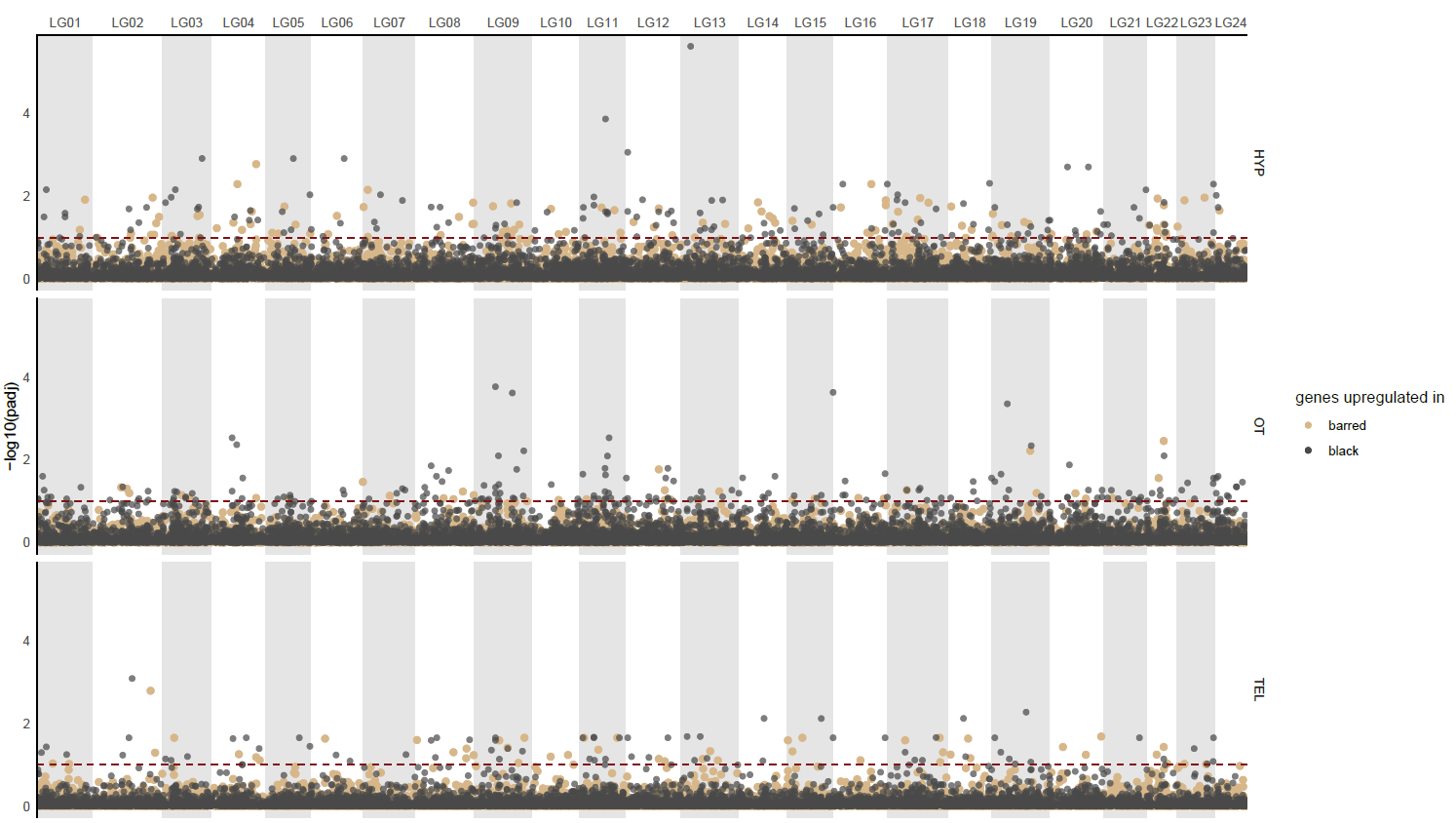


**Supplementary Figure S5:** Adjusted p-values (padj) of all genes plotted along the genome as -log10 values. Genes with higher expression in barred or black hamlets are plotted in brown and grey, respectively. A dashed line at 1 indicates the significance threshold. Genes are plotted separately for each brain region.


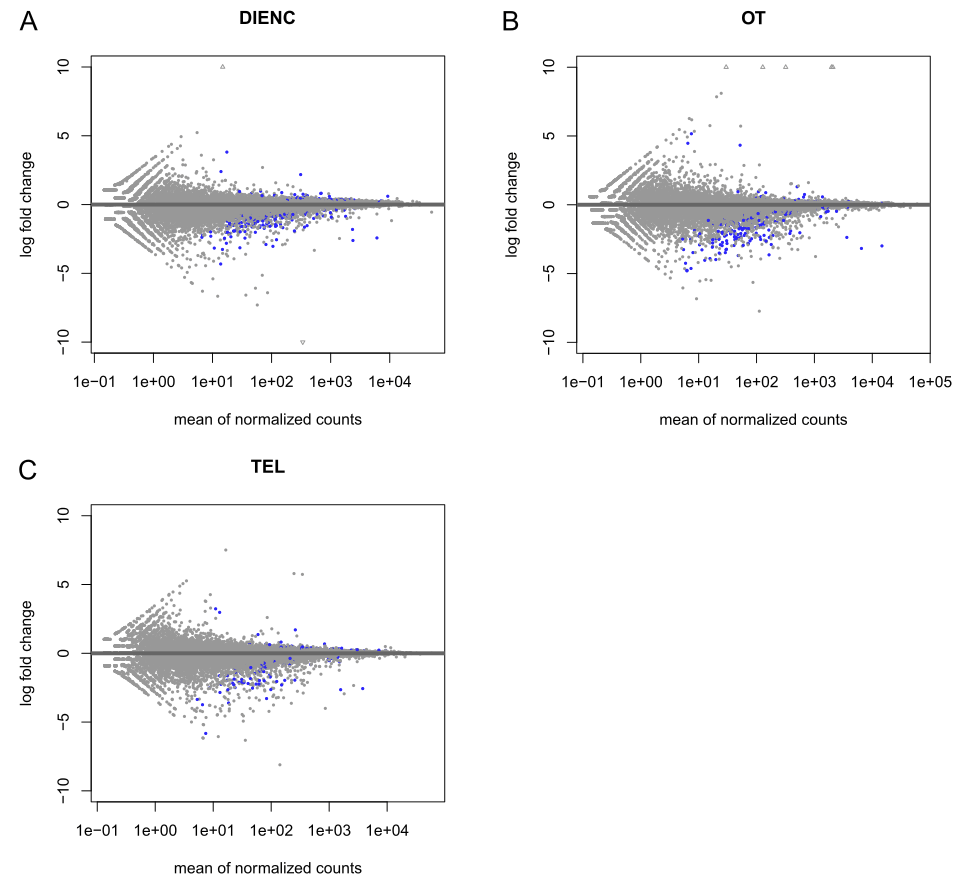


**Supplementary Figure S6:** MA plot for differential gene expression within the diencephalon (A), optic tectum (B) and telencephalon (C). Log fold change (LFC) values show the difference in mean expression between black and barred hamlets with higher expression in black hamlets for negative LFC and higher expression for barred hamlets for positive LFC. Each dot represents the expression of one gene with the mean of normalised gene counts on the x-axis showing highly expressed genes on the right sight of the axis and lowly expressed genes on the left side of the axis. Blue coloured dots indicate significantly differentially expressed genes.


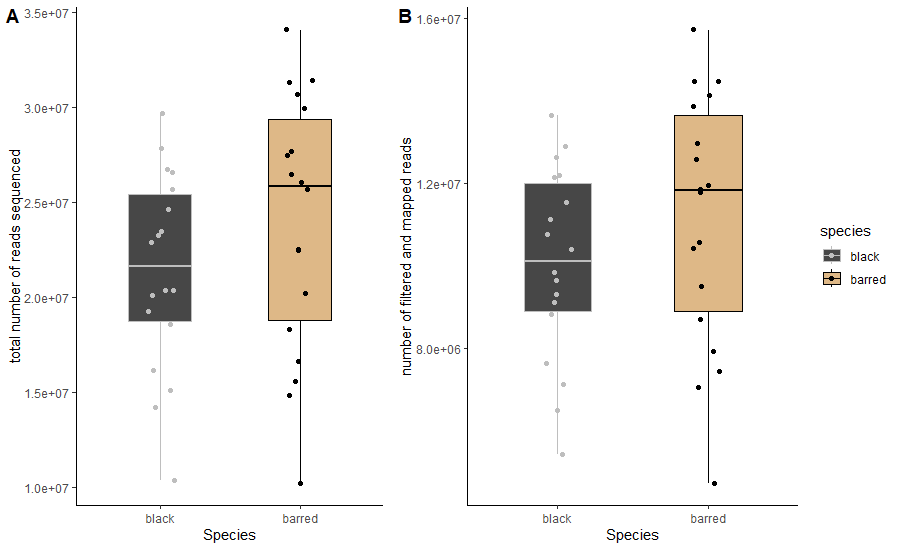


**Supplementary Figure S7:** Total number of raw reads (A) and number of filtered and uniquely mapped reads (B) per species. Boxes show the median with 25th and 75th percentiles. Whiskers indicate values within 1.5 times the interquartile range, and each dot represents one sample. Test statistics: (A) Total read counts: ANOVA, F 1,34 = 1.629, P = 0.21, mean ± sd: barred = 23.96 ± 6.77 Mio, black = 21.39 ± 5.2 Mio; (B) uniquely mapped reads: ANOVA, F 1,34 = 1.427, P = 0.241, mean ± sd: barred = 11.13 ± 3.05 Mio, black = 10.05 ± 2.33 Mio. Percentage reads uniquely mapped: barred: 46.6 ± 0.8 %, black: 47.2 ± 1.6 %.


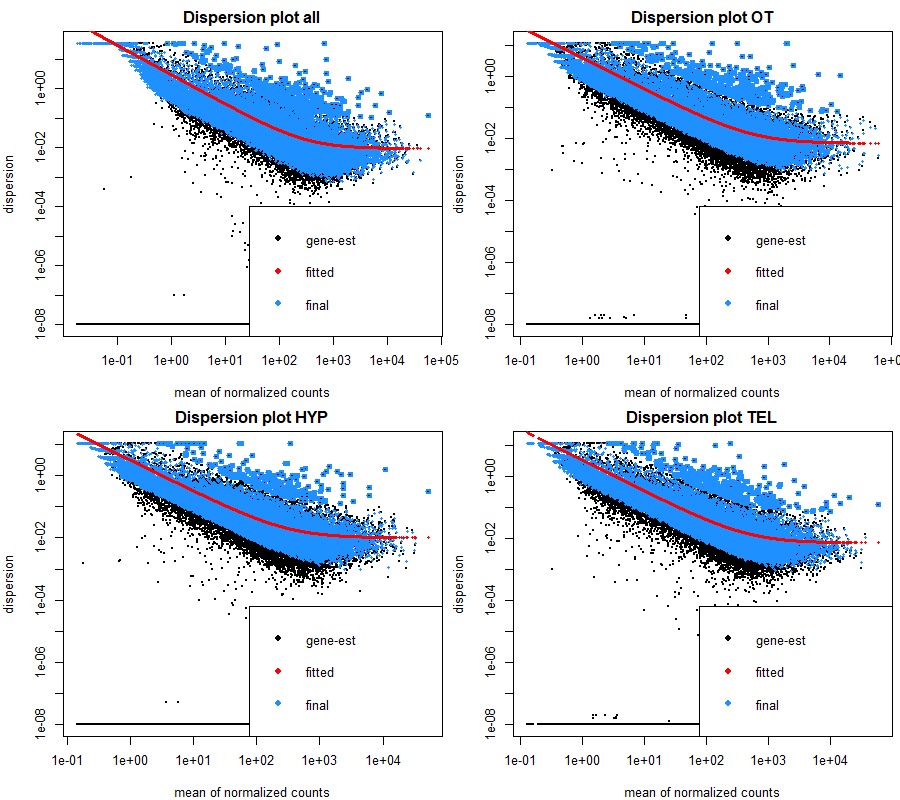


**Supplementary Figure S8:** Dispersion plot generated within the DESeq2 analyses pipeline for all data (A) and separately for the three brain regions: (B) optic tectum, OT; (C) diencephalon (HYP); (D) telencephalon (TEL). The plots display the gene-wise dispersion estimates (black dots), the fitted dispersion trend line (red), and the final dispersion estimates used for differential expression analysis (blue dots) with the mean normalised count for each gene on the x-axis and the dispersion value on the y-axis.
