# Supplementary material for: Differences in Colour Pattern, Behaviour and Gene Expression in the Brain Suggest Divergent Camouflage Strategies in Sympatric Reef Fish Species
Source: Mol Ecol. 2025 Apr 29;34(11):e17748. doi: 10.1111/mec.17748 (PMC12100586; doi:10.1111/mec.17748)
Supplement: Supplementary file 6 — Table S5. Highlighted brain transcriptomic results. A list of genes consistently differentially expressed across all three brain regions. For a list of all DEGs see Table S4. [file MEC-34-e17748-s003.docx]

| Gene ID | Gene Name | Description | Direction |
| --- | --- | --- | --- |
| HYPPUEv3G00000080054 | RXFP1 | Relaxin receptor 1 | up in barred |
| HYPPUEv3G00000168446 | KIFAP3 | Kinesin-associated protein 3 | up in barred |
| HYPPUEv3G00000247145 | DNASE1L1 | Deoxyribonuclease-1-like 1 | up in barred |
| HYPPUEv3G00000304080 | UNC79 | Protein unc-79 homolog | up in barred |
| HYPPUEv3G00000532634 | PCDHA7 | Protocadherin alpha-7 | up in barred |
| HYPPUEv3G00000148939 | RTP3_1 | Receptor-transporting protein 3 | up in black |
| HYPPUEv3G00000215956 | HBB1_1 | Hemoglobin subunit beta-A | up in black |
| HYPPUEv3G00000215964 | HBAB | Hemoglobin subunit alpha-B | up in black |
| HYPPUEv3G00000222347 | IL6ST | Interleukin-6 receptor subunit beta | up in black |
| HYPPUEv3G00000232882 | DNASE1L3 | Deoxyribonuclease gamma | up in black |
| HYPPUEv3G00000251214 | TRAF3IP3 | TRAF3-interacting JNK-activating modulator | up in black |
| HYPPUEv3G00000323021 | AT5G56560 | Putative FBD-associated F-box protein At5g56560 | up in black |
| HYPPUEv3G00000343944 | NA | NA | up in black |
| HYPPUEv3G00000388395 | NA | NA | up in black |
| HYPPUEv3G00000510143 | CLDN20 | Claudin-20 | up in black |
| HYPPUEv3G00000520954 | NLRP12 | NACHT, LRR and PYD domains-containing protein 12 | up in black |

**Supplementary Table S5:** A list of genes consistently differentially expressed across all three brain regions. For a list of all DEGs see Supplementary Table S4.
